# Treatment of Strip Perforation Using Root MTA: A Case Report

**Published:** 2013-05-01

**Authors:** Mohammad Froughreyhani, Amin Salem Milani, Behnaz Barakatein, Vahhab Shiezadeh

**Affiliations:** 1 Department of Endodontics, Dental School, Tabriz University of Medical Sciences, Tabriz, Iran; 2 Dental and Periodontal Research Center, Tabriz University of Medical Sciences, Tabriz, Iran; 3 Department of Endodontics, Dental School, Isfahan University of Medical Sciences, Isfahan, Iran; 4 Dental School, Zanjan University of Medical Sciences, Zanjan, Iran

**Keywords:** Lateral Root Perforation, Mandibular, Molar, Mineral Trioxide Aggregate, Root MTA

## Abstract

Root perforations are an undesired complication of endodontic treatment which result in loss of integrity of the root, and adversely affect the prognosis of the treatment. Recently, Iranian mineral trioxide aggregate [Root MTA] has been introduced as an ideal material for perforation repair. In this article a successful repair of strip root perforation of mandibular molar using Root MTA is presented with 15-month follow-up. This case suggests that Root MTA may be a substitute material for the treatment of strip perforation; however, more clinical studies with larger sample size and longer follow-ups are needed.

## Introduction

Root perforations may occur during access cavity preparation, root canal instrumentation, post space preparation, or as a result of the extension of internal resorption into the periradicular tissues. They result in loss of integrity of the root and destruction of the adjacent periodontal tissues [1]. Root perforation can be repaired through the access cavity or by surgical intervention. When repair of a perforation fails after an intracanal approach or if the perforation site is inaccessible through the access cavity, surgical repair is indicated [2]. Prognosis of perforation repair mostly depends on elimination and prevention of infection of the perforation site. In addition, the use of a biocompatible material that seals the perforation will limit periodontal inflammation [3].

Different materials have been used for perforation repair, including amalgam, IRM, Super EBA, Cavit, gutta-percha, glass ionomer, resin-ionomer, new generation dentin-enamel bonding systems, and composites; but none of them fulfill the criteria of an ideal repair material that include sealing ability, biocompatibility, and ability to induce osteogenesis and cementogenesis [4].

Mineral trioxide aggregate [MTA] is a widely known material that was originally proposed to repair perforations; however, it gradually gained a variety of clinical applications such as root end filling [5, 6], pulp capping in primary and permanent teeth [7, 8], apical barrier for immature permanent teeth [9], and repair of tooth resorption [10]. MTA has also shown strengthening effect on dentinal wall of immature roots [11, 12] and was also proposed as a suitable coronal barrier material [13]. MTA is primarily composed of tricalcium silicate, tricalcium aluminate, tricalcium oxide, and silicate oxide that upon hydration, forms a colloidal gel that solidifies in approximately 3h [14]. MTA has shown many favorable properties including a good sealing, biocompatibility, antibacterial effect, radiopacity, and ability to set in the presence of blood [15-19]. In vitro perforations repaired with MTA show significantly less leakage when compared with amalgam, IRM, ZOE, and Super EBA [20, 21]. Its ability to induce osteogenesis and cementogenesis make MTA a suitable material for the treatment of root perforations [22]. The original commercial brand of MTA is ProRoot MTA that was developed by Tulsa Dental. The high cost of ProRoot MTA persuaded the researchers to develop other brands with lower costs.

Root MTA was developed by Lotfi in Tabriz University of Medical Sciences, [Iran] as a less expensive brand of MTA. Studies have shown favorable properties of Root MTA [23, 24]. There are still not enough case reports and clinical studies demonstrating the clinical applications of Root MTA. This study presents a mandibular molar with iatrogenic stripping perforation which was successfully managed using Root MTA.

## Case Report

A 25-year-old woman with the complaint of persistent drainage of pus in the right mandibular vestibule region was referred to the Endodontic Department of Tabriz Dental School. Patient’s medical history revealed no significant findings.

**Figure 1.**
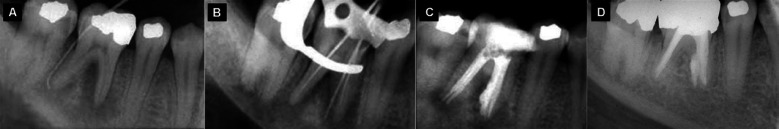
A] preoperative tracing radiograph showing large furcal and periapical lesions at the mesial and distal roots of the mandibular first molar; B] a strip perforation on the distal aspect of the mesiolingual canal; C] repair of the perforation with Root MTA; slight extrusion of the material is evident; D] 15-month Follow-up radiograph showing complete resolution of periapical and furcal lesions.

On clinical examination, the right mandibular first molar was sensitive to percussion; however, probing depth and mobility were within normal range. Right mandibular first molar showed no response to thermal and electric pulp testing. Tracing with gutta-percha pointed to the right first mandibular molar as the origin of the draining sinus tract. Radiographic examination also revealed furcal and apical radiolucency [Figure 1A]. Diagnosis of pulp necrosis with chronic periradicular periodontitis was established. After isolation with rubber dam, standard access cavity was prepared, and canal instrumentation was started. The instrumentation was stopped because of sudden bleeding in mesiolingual canal of mesial root. Stripping perforation of the mesial root was seen on the intraoral periapical radiograph [Figure 1B]. The patient was immediately referred to the post-graduate section of the Endodontic Department. There, the instrumentation of the canals was continued using ProTaper Universal [Dentsply Maillefer, Ballaigues, Switzerland] in a crown down approach except in the mesiolingual canal to prevent increasing the size of perforation. One percent sodium hypochlorite [NaOCl] was used as irrigant during instrumentation. Because of persistent hemorrhage, a calcium hydroxide dressing was placed into the canals, and the tooth was temporary restored with Zonalin [Purton, Wiltshin, Sweden]. The patient was asymptomatic between appointments. At the subsequent visit 7 days later, the tooth was isolated, temporary restoration was removed, and the canals were thoroughly irrigated with 1% NaOCl. The canals were then gently dried and obturated with gutta-percha [Gapadent, Hamburg, Germany] and AH26 sealer [Dentsply DeTrey, Konstanz, Germany] using lateral compaction method. The gutta-percha coronal to the perforation site in mesiolingual canal was removed. Root MTA [Salamifar, Tehran, Iran] was mixed according to the manufacturer’s instructions and compacted into the coronal section of the canal and perforation site using Schilder plugger [Dentsply Caulk, Milford, USA] [Figure 1C]. A wet cotton pellet was placed over the MTA, and the tooth was temporary restored using IRM [Dentsply, York, PA, USA]. After 48 hours, the temporary restoration and cotton pellet were removed, and the tooth was permanently restored with amalgam [Synalloy, Dentoria, France]. On 15-month recall, the patient had no signs or symptoms, and radiographic examination demonstrated complete resolution of furcal and periapical radiolucency [Figure 1D].

## Discussion

Strip perforation is an unwanted procedural accident that negatively affects the prognosis. Mesiobuccal root of maxillary molars and the mesial root of mandibular molars are highly susceptible to strip perforation because of thin dentinal walls. Inappropriate instrumentation as well as large instruments during preparation in these thin root canals can cause strip perforation. Mechanical trauma to periodontal tissue as a result of perforation and possible contamination of the site often lead to inflammation and osseous destruction. Strip perforation differs from other perforations because of its large affected area, irregular edge of the perforation site, and difficulty in sealing the perforation [3]. One of the most important factors affecting the prognosis is the time elapsed since the occurrence of the perforation [1, 25]. In the present case, the time between perforation and repair was short; this contributed to the success of the treatment. In this case, the canals were treated with calcium hydroxide between appointments to reduce exudation, excessive bleeding, and inflammation of the periradicular tissues before the obturation of the canals and sealing of the perforation with MTA. Studies have shown that calcium hydroxide eliminates bacteria and reduces inflammation and bleeding from the perforation site [26, 27]. Application of calcium hydroxide in this case reduced inflammation and bleeding, making the treatment less complicated. To prevent possible negative effect of calcium hydroxide on setting of MTA [28], the mesiolingual canal was thoroughly irrigated prior to MTA placement.

Mineral trioxide aggregate is well known biocompatible material that induces cementogenesis around the perforation sites [29]. It has been used successfully to repair the perforations in different clinical situations [15, 18, 27, 30]. Because of hydrophilic nature of MTA, moisture is required for complete setting of the material [4]. However, some studies have proposed that the moisture from the tissue side is often enough for proper setting of MTA without the need for additional moisture from within the canal [31, 32]. We used a wet cotton pellet for 48 h to assure proper setting of MTA.

Root MTA is Iranian brand of mineral trioxide aggregate produced by Dr. M. Lotfi. The studies on microleakage, cytotoxicity, and biocompatibility of Root MTA showed favorable results similar to the original ProRoot MTA [23, 24, 33-35].

On 15-month recall, the repaired tooth was clinically and radiographically healthy and continued to satisfy functional demands. This case presented successful treatment of strip perforation using Root MTA. We may conclude that Root MTA is a suitable material for perforation. However, further clinical studies are still required to determine the prognosis of root perforations repaired with Root MTA in long term.
